# Safety and Efficacy of Fixed-Dose Pantoprazole and Sustained Release Levosulpiride for Short-Term Management of Gastroesophageal Reflux Disease

**DOI:** 10.14740/gr2117

**Published:** 2026-08-26

**Authors:** Yogesh Garje, Vijay Kamat, Kalidas Biswas, M. Murugesh, Manoj Kumar Agarwal, Pushparaj Karmarkar, Narendra D. Kulkarni, Jitendra Anand, Sunil Kumar Mahavar, B. Ramesh Kumar, Mukesh Mishra, P. Shravan Kumar, Ashwini Satpathy, Shruti Dharmadhikari, Chintan Khandhedia, Neeraj Markandeywar, Amey Mane, Suyog Mehta

**Affiliations:** a (Ex employee) Medical Affairs and Clinical Research, Sun Pharma Laboratories Limited, Mumbai 400063, Maharashtra, India; b Department of Surgery, Karnataka Institute of Medical Sciences, Hubli 22, Dharwad, Karnataka, India; c Department of Gastroenterology, Medical College & Hospital, Kolkata 700037, West Bengal, India; d Clinical Research Room, Kovai Diabetes Speciality Centre and Hospital, Coimbatore 641009, Tamil Nadu, India; e Department of Gastroenterology, Belle Vue Clinic, Kolkata 700017, West Bengal, India; f Department of General Surgery, Sanjeevan Hospital, Pune 411004, Maharashtra, India; g Dr. Hedgewar Hospital, Aurangabad 431005, Maharashtra, India; h Kanoria Hospital & Research Centre, Gandhinagar 382428, Gujarat, India; i Department of Medicine, S.M.S. Medical College & Attached Hospital, Jaipur 302004, Rajasthan, India; j Department of Gastroenterology, Osmania General Hospital, Hyderabad, Telangana, India; k Department of Internal Medicine, Shat Ayu Multispeciality Hospital, Nagpur 440012, Maharashtra, India; l Gastroenterology Department, Gandhi Hospital, Secunderabad, Telangana, India; m Medical Affairs and Clinical Research, Mankind Pharma, New Delhi, India; n Medical Affairs and Clinical Research, Sun Pharma Laboratories Limited, Mumbai 400063, Maharashtra, India

**Keywords:** GERD, Pantoprazole, Levosulpiride, FSSG, Likert Scale, CGI-I, CGI-S, MSAS

## Abstract

**Background:**

Gastroesophageal reflux disease (GERD) and dyspepsia are highly prevalent in India. Pantoprazole suppresses gastric acid secretion by inhibiting H^+^, K^+^-ATPase enzyme, yet patients experience inadequate symptom control with proton pump inhibitor (PPI) monotherapy. Levosulpiride, a selective dopamine D2 receptor antagonist, enhances gastrointestinal motility and visceral sensitivity, offering additional symptomatic relief. The aim of the study was to evaluate the safety and efficacy of a fixed-dose combination (FDC) of pantoprazole sodium 40 mg and levosulpiride sustained-release 75 mg for short-term therapy of GERD in patients unresponsive to PPI monotherapy.

**Methods:**

This phase IV, open-label, single-arm study was conducted at 11 sites across India. Of 540 screened patients, 509 were enrolled and received FDC once daily for 4 weeks. Safety was evaluated through adverse events (AEs) and serious AEs (SAEs). Efficacy was assessed using the Frequency Scale for the Symptoms of GERD (FSSG), Likert Scale, Clinical Global Impression of Improvement (CGI-I), Clinical Global Impression of Severity (CGI-S), and Modified Simpson Angus Scale (MSAS).

**Results:**

Of 509 patients, 492 completed the study. Seventy-nine AEs were documented in 69 patients, all mild-to-moderate and no SAEs were reported. The FDC demonstrated significant reduction in GERD symptom severity, with a mean (standard deviation) change from baseline of −16.99 (8.76) (P < 0.0001) on FSSG Scale at visit 4. Additionally, reductions were observed in acid reflux symptoms with a mean (SD) change from baseline of −9.15 (5.25) and dyspeptic symptoms −7.8 (4.05); both changes were statistically significant (P < 0.0001). The Likert Scale also demonstrated a significant reduction in symptom severity indicated by a mean (standard deviation) change from baseline of −1.26 (1.00) (P < 0.0001). At visit 4, CGI-I scores classified 196 patients as “very much improved”, while CGI-S indicated “normal” status in 173 patients. The absence of significant movement disorders, assessed by MSAS at visit 4, further supports the favorable safety profile of FDC in management of GERD.

**Conclusion:**

The FDC of pantoprazole and levosulpiride is well tolerated and effective for the short-term treatment of GERD in patients unresponsive to PPI therapy. Symptom reductions and favorable global assessments indicate therapeutic benefit of therapy in refractory GERD population.

## Introduction

Gastroesophageal reflux disease (GERD) is a condition characterized by the backward flow of gastric contents into the esophagus, leading to symptoms such as heartburn and regurgitation [1]. In some patients, GERD may also present with extraesophageal manifestations, including chronic cough, asthma, or occasional mucosal changes [2]. The exact etiology of GERD remains unclear; however, its pathogenesis involves multiple factors, including esophagogastric junction dysfunction, esophageal hypersensitivity, impaired esophageal bolus transit, and elevated intragastric pressure [3]. Additionally, various risk factors contribute to the development of GERD, including age, obesity, lifestyle choices, and the use of nonsteroidal anti-inflammatory drugs (NSAIDs) [4].

According to the global age-standardized prevalence rate (ASPR), the number of GERD cases worldwide reached 783.9 million in 2019, with the highest incidence reported in the USA, China, and India [5]. In India, the prevalence of GERD ranges from 7.6% to 30%. Most population-based studies report rates below 10%, while cohort studies indicate higher rates [6].

Treatment options for GERD focus on managing and reducing symptoms along with the patient’s quality of life [7]. The first-line therapy for the medical management of GERD is acid suppression which includes proton pump inhibitors (PPIs), antacids, muscarinic antagonists, and lifestyle modifications [8, 9]. Pantoprazole, a PPI, is used to reduce gastric acid production by the inhibition of the H^+^, K^+^-ATPase enzyme in the stomach lining [10]. The interaction of PPIs with the sulfhydryl groups of cysteine residues within the enzyme induces conformational changes that lead to its inactivation, resulting in a reduction in acid secretion [11].

Although PPIs demonstrate long-term effectiveness and notable healing rates [12], studies have reported varying rates of intragastric pH control and symptomatic relief in short-term therapies [13]. Furthermore, PPIs alone do not address underlying gut motility issues or lower esophageal sphincter tone [14]. According to Gastroenterology Expert Review (2021), up to 40% of GERD patients exhibit a suboptimal response to PPI monotherapy, with persistent symptoms despite standard PPI treatment [15, 16]. In these cases, GERD can be effectively managed with combination therapy (PPI with prokinetic agents), reducing acid levels, or increasing PPI dosages [12, 17]. The synergistic approach of prokinetic agents enhances the bioavailability of PPI, reduces acid production, boosts lower esophageal sphincter tone, and leads to improved clinical outcomes in GERD patients [10, 18]. Among prokinetics, levosulpiride acts by antagonizing D2 dopamine receptors in the gastrointestinal tract, thereby enhancing lower esophageal sphincter (LES) pressure and accelerating gastric emptying. These pharmacological effects help reduce the severity of acid reflux and make levosulpiride an effective adjunctive therapy in GERD patients who are unresponsive to PPI therapy [19, 20].

The growing need for alternative treatment strategies for GERD highlights the importance of combination therapy using PPIs and prokinetic agents. This approach is promising because it simultaneously reduces gastric acid levels and enhances gastrointestinal motility. Therefore, the present study was planned to evaluate the safety and efficacy of a fixed-dose combination (FDC) of pantoprazole sodium 40 mg and levosulpiride sustained-release (SR) 75 mg in managing GERD in patients who are unresponsive to PPI monotherapy.

## Materials and Methods

### Study design

This phase IV, open-label, single-arm, multicentric study was conducted at 11 sites across India between March 2016 and May 2017. The study sites were Gandhi Hospital (Hyderabad), Medical College and Hospital (Kolkata), Belle Vue Clinic (Kolkata), Karnataka Institute of Medical Sciences (Dharwad), Dr Hedgewar Hospital (Aurangabad), Kanoria Hospital and Research Centre (Gandhinagar), Kovai Diabetes Specialty Centre and Hospital (Coimbatore), Osmania General Hospital (Hyderabad), S.M.S College and Attached Hospital (Jaipur), Sanjeevan Hospital (Pune), and Shat Aayu Multispecialty Hospital (Nagpur). The study received approval from the Institutional Ethics Committee/Institutional Review Board (IEC/IRB) and was registered with the Clinical Trials Registry-India (CTRI/2016/03/006730). The study was conducted in accordance with the principles of the ICH-GCP, applicable local laws, regulatory requirements, and ethical standards including the Declaration of Helsinki. All patients provided informed consent prior to participating in the study.

### Study participation

This study included male and female patients aged 18 to 65 years with a history of GERD within the last 3 months who had received PPI monotherapy for 4 weeks prior to screening but remained inadequately controlled. Refractory GERD was defined as persistence of predominant symptoms (heartburn and/or regurgitation) despite adequate PPI therapy. The exclusion criteria were: pregnant, lactating, or non-contraceptive-using women of childbearing age; patients with peptic ulcer, Zollinger–Ellison syndrome, or a history of gastrointestinal surgery (excluding appendectomy, cholecystectomy, and polypectomy); those with scleroderma, pancreatitis, cancer, Parkinson’s disease, epilepsy, mania, inflammatory bowel disease, severe illnesses (hepatic, renal, cardiac), uncontrolled diabetes, hypertension, clotting disorders; heavy smokers (> 10 cigarettes per day in last 6 months); individuals with hypersensitivity or contraindications to pantoprazole or levosulpiride; recent drug dependence or abuse (within 2 years); and those unwilling or unable to comply with the study protocol.

### Study plan

**Figure 1:**
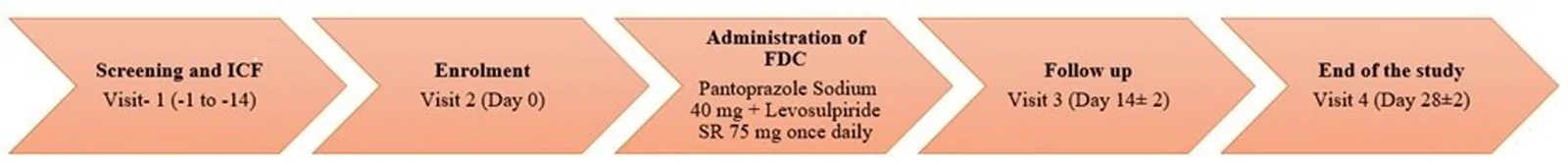
Study design.

This study aimed to evaluate the safety and efficacy of an FDC of pantoprazole sodium 40 mg and levosulpiride 75 mg (SR) in GERD patients who do not respond to PPI monotherapy. The recommended dosage of FDC was administered as one capsule once daily in the morning before breakfast for 28 days. The duration of study participation of each patient was up to 6 weeks (2 weeks for screening and 4 weeks for treatment). The study was scheduled for four visits: visit 1: screening visit (day −14 to day −1), visit 2: enrollment visit (day 0), visit 3: follow-up visit (day 14 ± 2), and visit 4: end of the study (day 28 ± 2) (Fig. 1).

Endoscopic assessments were performed during the screening and end of the study. Blood and urine samples were collected and analyzed at the screening and end of the study. Patients were requested to discontinue their existing PPI medication and switch to the study drug. Each patient received a once-daily dose of the FDC capsule before breakfast for 4 weeks. Urine samples underwent routine and microscopic examinations, including pregnancy tests for female patients.

### Study endpoints

The primary endpoint was the evaluation of the proportion of patients who had adverse events (AEs) and serious AEs (SAEs) from visit 2 (baseline) to visit 4 (end of the study), during 4 weeks of treatment. The secondary endpoints assessed the efficacy of FDC using Frequency Scale for the Symptoms of GERD (FSSG), the Likert Scale, the Clinical Global Impression of Improvement (CGI-I), and the Clinical Global Impression of Severity (CGI-S) at visits 2 and 4. Extrapyramidal symptoms were evaluated using the Modified Simpson Angus Scale (MSAS) at visits 2 and 4.

### Sample size and statistical analysis

The regulatory authority recommended to conduct the study in at least 500 GERD patients who do not respond to PPI alone. Descriptive statistics for continuous and ordinal variables, including the mean and standard deviation (SD), were calculated based on the available data at respective visits. Categorical variables, such as gender and AEs, were summarized using counts and percentages. All P-values were unadjusted for multiple comparisons, and the results were reported as P-values with approximate 95% confidence intervals (CIs). All statistical tests were conducted using SAS version 9.1.3.

## Results

### Patient demographics

Of the 540 patients screened, 509 eligible patients were enrolled in the study. There were 304 (59.7%) males and 205 (40.3%) females. The population had a mean (SD) age of 39.06 (11.92) years and a mean body mass index (BMI) of 24.15 (3.69) kg/m^2^. At screening, 250 (49.1%) patients were taking pantoprazole, 112 (22%) patients were taking omeprazole, 87 (17.1%) patients were taking rabeprazole, 42 (8.3%) patients were taking esomeprazole, and 18 (3.5%) patients were taking lansoprazole.

### Patient disposition

**Figure 2:**
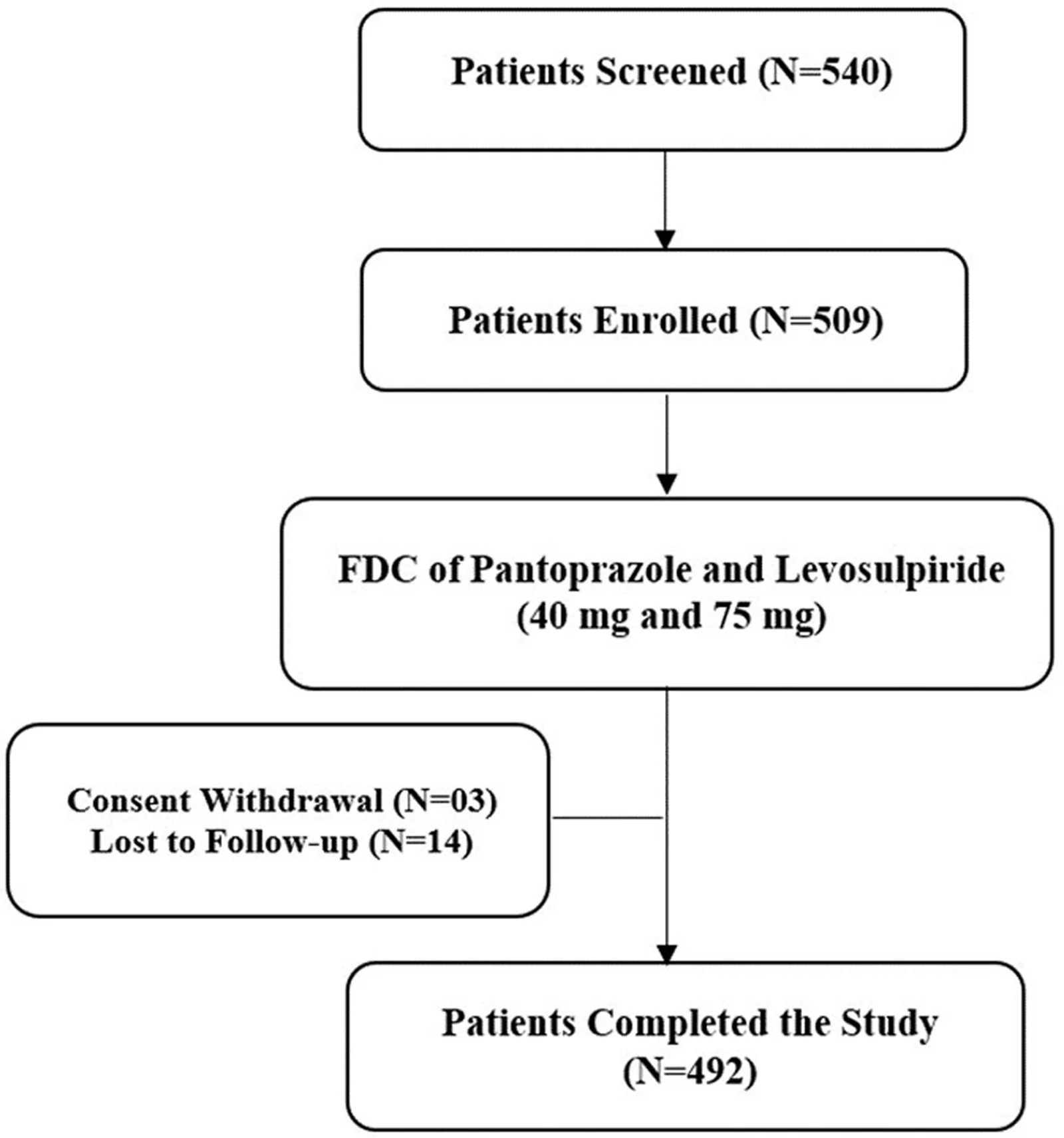
Patient disposition.

Of the 509 patients enrolled, 492 (96.7%) completed the study, while 17 (3.3%) patients discontinued, including three (0.6%) patients who withdrew their consent, and remaining 14 (2.8%) patients were lost to follow-up (Fig. 2).

### Safety analysis

Of the 509 patients enrolled, 69 (13.6%) experienced 79 AEs. Of these 79 AEs, 26 were mild and 53 were moderate in severity. The most prevalent AEs were increased blood prolactin levels followed by headache, pyrexia, diarrhea, and urinary tract infections. Elevated serum prolactin levels were observed in 50 patients (9.8%), comprising 10 (2.0%) mild and 40 (7.9%) moderate (Table 1).

**Table 1:** Incidence of Treatment-Emergent Adverse Events by System Organ Class and Preferred Term

System organ class: preferred term	FDC of pantoprazole sodium + levosulpiride SR capsules (N = 509)	Severity of adverse events	
		Mild	Moderate
Abdominal pain	1 (0.2%)	1 (0.2%)	0
Constipation	1 (0.2%)	1 (0.2%)	0
Diarrhea	3 (0.6%)	2 (0.4%)	1 (0.2%)
Nausea	2 (0.4%)	2 (0.4%)	0
Pyrexia	5 (1%)	1 (0.2%)	4 (0.8%)
Nasopharyngitis	1 (0.2%)	1 (0.2%)	0
Rhinitis	2 (0.4%)	0	2 (0.4%)
Urinary tract infection	3 (0.6%)	0	3 (0.6%)
Blood prolactin increased	50 (9.8%)	10 (2%)	40 (7.9%)
Dizziness	1 (0.2%)	1 (0.2%)	0
Headache	6 (1.2%)	4 (0.8%)	2 (0.4%)
Testicular pain	1 (0.2%)	1 (0.2%)	0
Cough	1 (0.2%)	0	1 (0.2%)
Oropharyngeal pain	1 (0.2%)	1 (0.2%)	0
Rhinitis allergic	1 (0.2%)	1 (0.2%)	0

FDC: fixed-dose combination; SR: sustained release.

### Efficacy analysis

#### Change in FSSG

**Figure 3:**
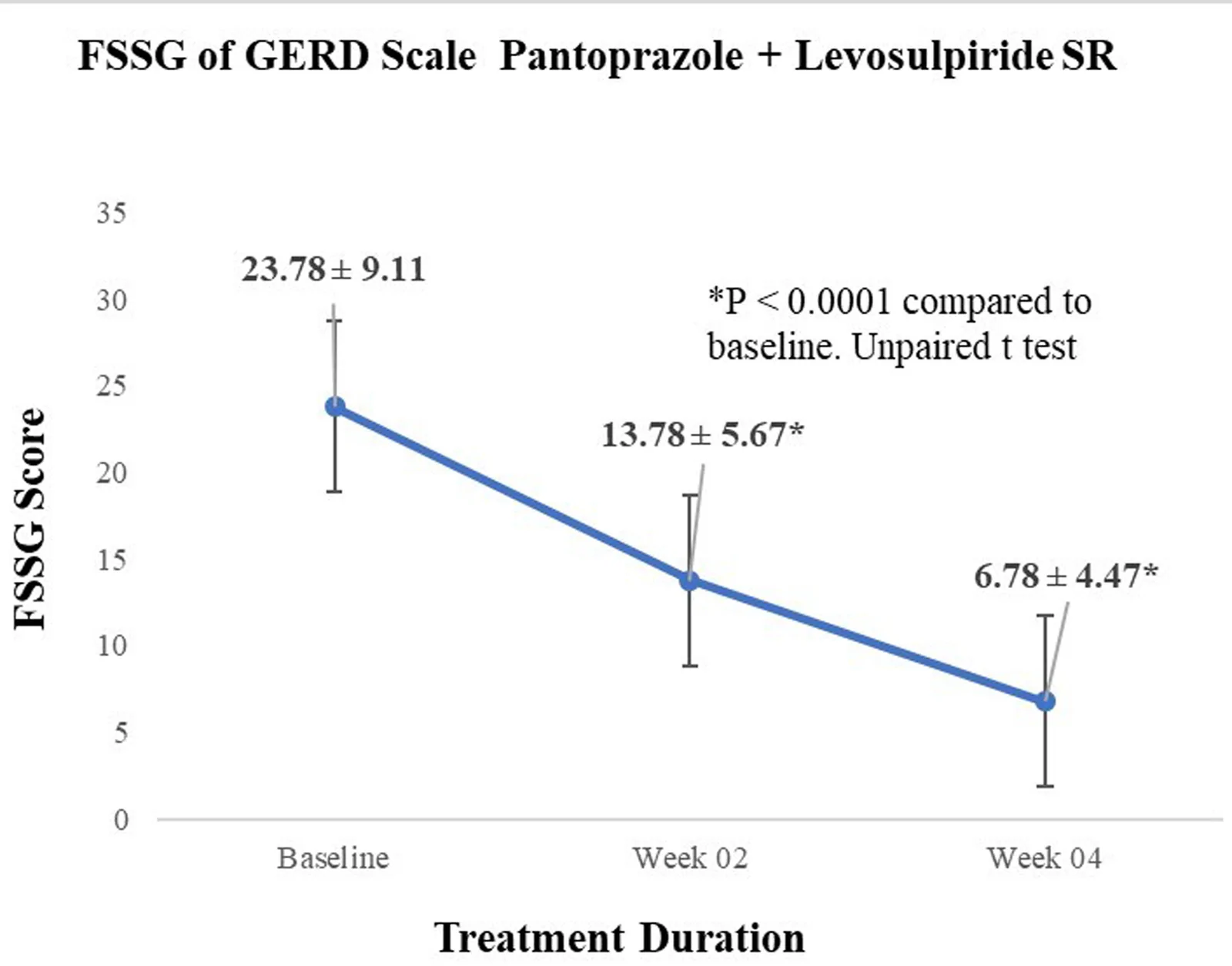
Change in Frequency Scale for the Symptoms of GERD Scale from baseline.

Patients achieved a significant reduction in the symptoms of GERD by visit 4 (Fig. 3). At visit 2, the mean (SD) FSSG was 23.78 (9.11), which significantly reduced to 6.78 (4.47) by visit 4, with a mean (SD) change of −16.99 (8.76) from baseline. This reduction was statistically significant (P < 0.0001), indicating an improvement in GERD symptoms.

**Figure 4:**
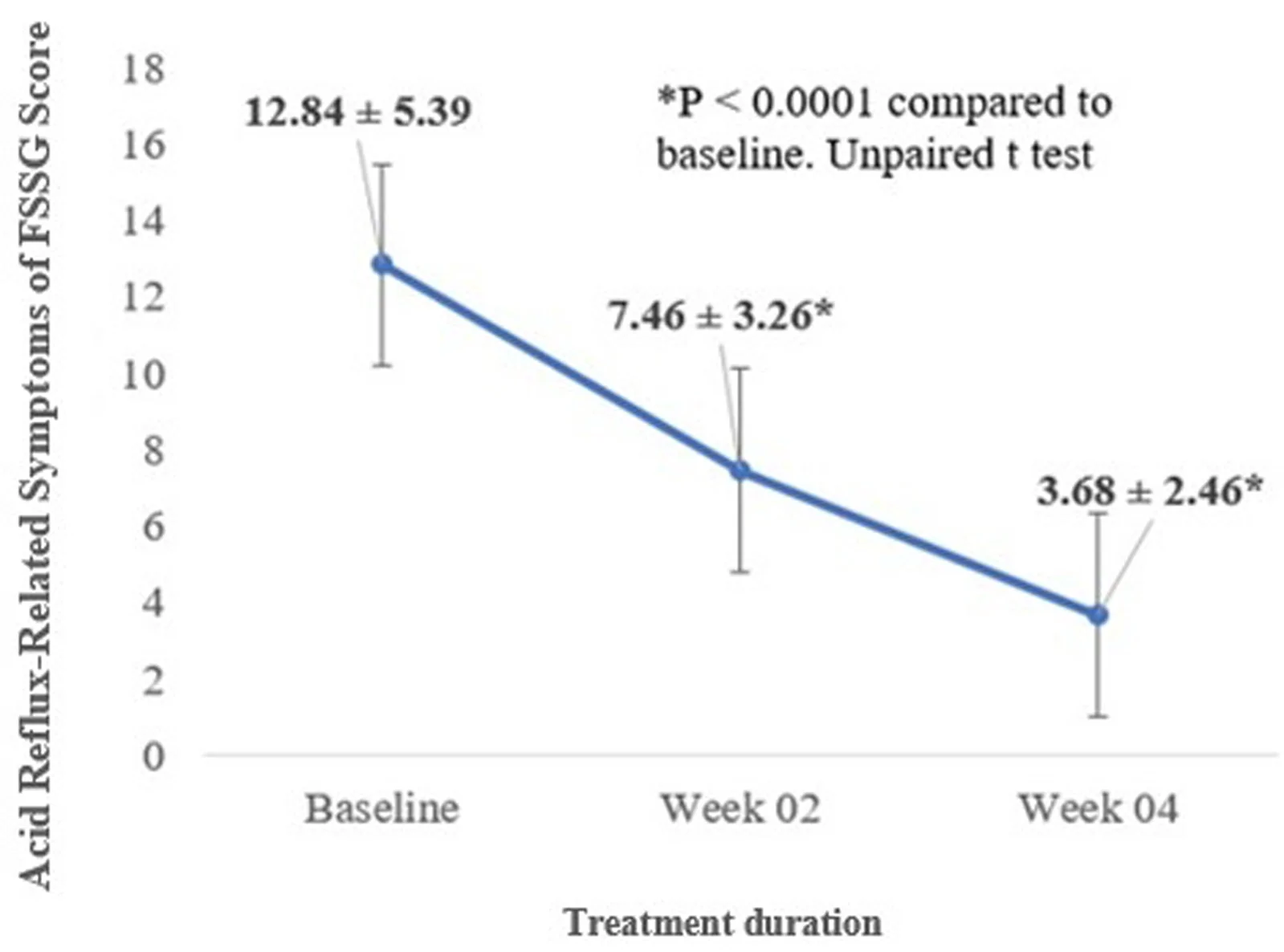
Acid reflux-related symptoms of Frequency Scale for the Symptoms of GERD.

A notable decrease in acid reflux-related symptoms was observed at visit 4 (Fig. 4) from baseline. At visit 2, the mean (SD) for acid reflux symptoms was 12.84 (5.39) which reduced to 3.68 (2.46) by visit 4. The mean (SD) change from baseline was −9.15 (5.25), with a corresponding P-value of < 0.0001, highlighting the effectiveness of the investigational product in alleviating acid reflux-related symptoms.

**Figure 5:**
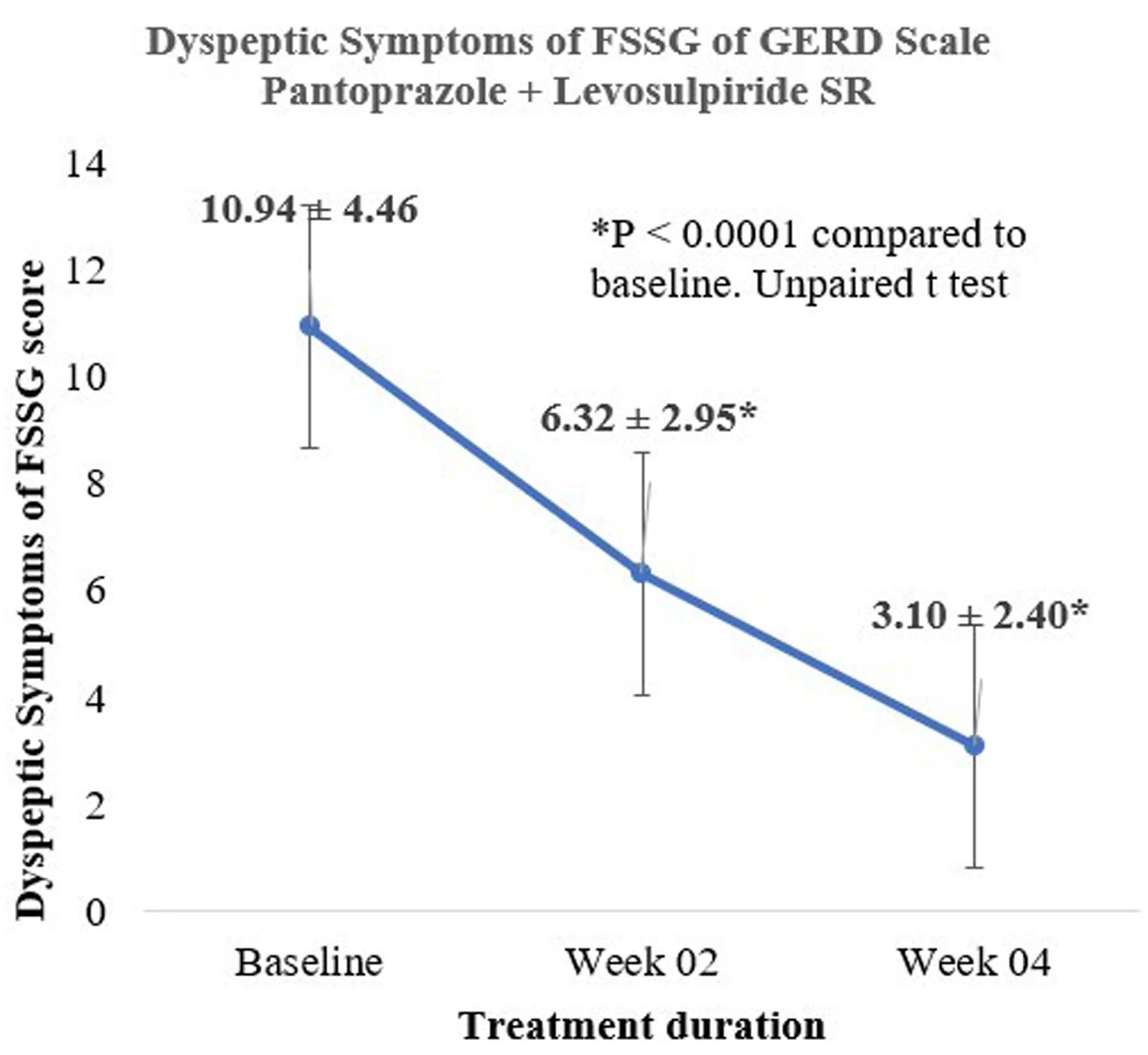
Dyspeptic symptoms of Frequency Scale for the Symptoms of GERD Scale from baseline.

A significant reduction in dyspeptic symptoms was observed at visit 4 (Fig. 5) from baseline. The mean (SD) for dyspeptic symptoms at visit 2 was 10.94 (4.46), which decreased to 3.10 (2.40) by visit 4. The mean (SD) change from baseline was −7.8 (4.05), with a P-value of < 0.0001. The results demonstrated the effectiveness of the investigational product in reducing dyspeptic symptoms.

#### Change in severity of symptoms using the Likert Scale

**Figure 6:**
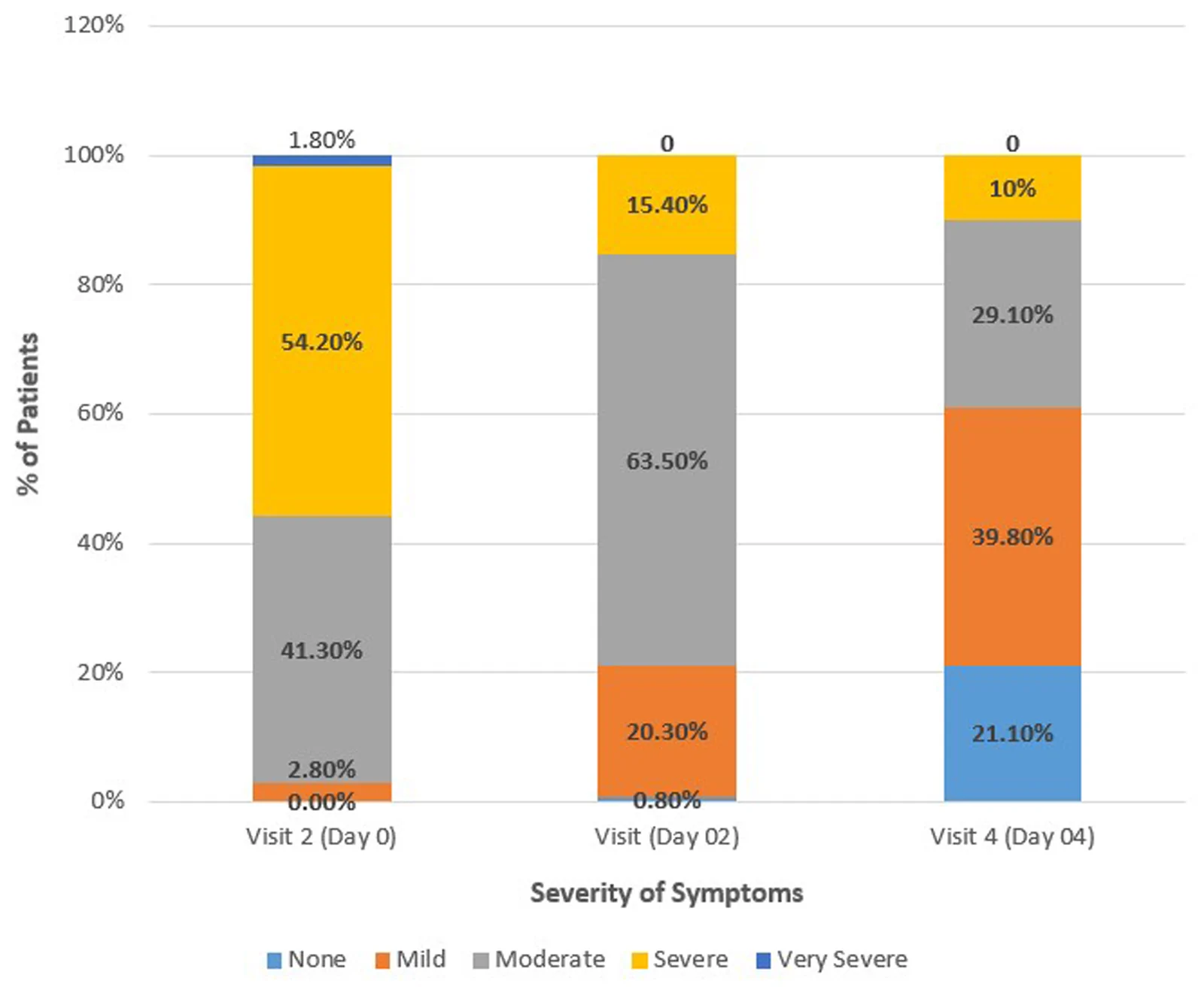
Change in severity of symptoms of GERD by Likert Scale.

The mean (SD) symptom severity score decreased from 2.55 ± 0.58 at baseline visit 2, to 1.93 ± 0.62 at visit 3, and further to 1.28 ± 0.91 by visit 4. The mean (SD) change from baseline was −0.62 (−22.8%) at visit 3 and −1.26 (−48%) by visit 4, both statistically significant with P-values < 0.0001, indicating a substantial reduction in GERD symptoms over the course of the study. Furthermore, at baseline (visit 2), the majority of patients reported moderate 210 (41.3%), severe 276 (54.2%), and very severe 9 (1.8%) symptoms. However, at visit 4, a notable improvement was observed, with moderate symptoms reported in 143 (29.1%) patients, and severe symptoms in 49 (10%) patients. None of the patients reported very severe symptoms (Fig. 6).

#### CGI-I

**Figure 7:**
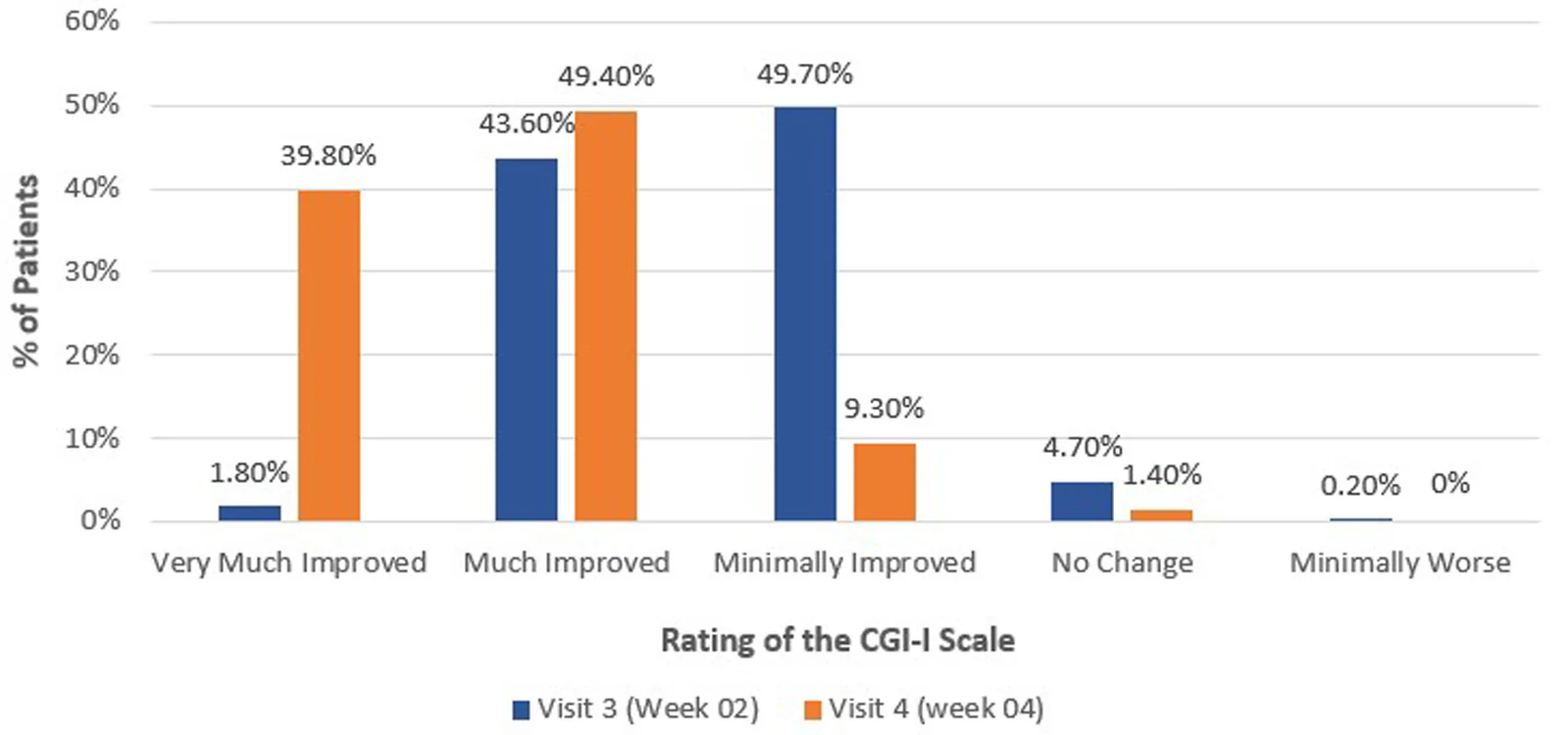
Change in Clinical Global Impression of Improvement (CGI-I).

A substantial improvement in GERD was observed at visit 4 compared to the baseline (Fig. 7). Most patients showed improvements by visit 4: 196 (39.8%) were classified as “very much improved”, 243 (49.4%) as “much improved”, 46 (9.3%) as “minimally improved”, and seven (1.4%) as “no change”.

#### Change in CGI-S

**Figure 8:**
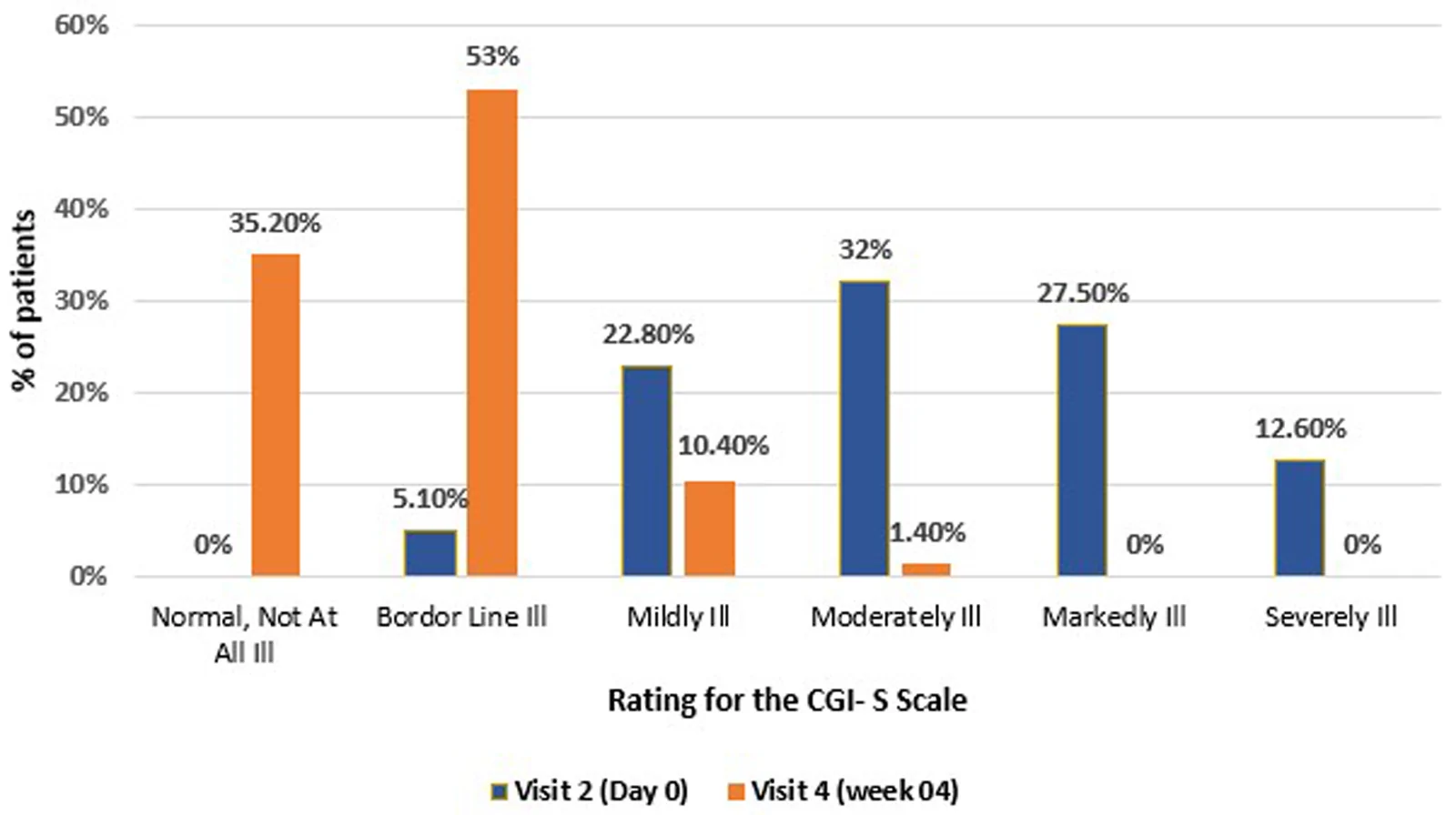
Change in Clinical Global Impression of Severity (CGI-S).

The reduction in severity scores was observed throughout the study. A significant increase in the number of patients categorized as “normal, not at all ill” notably increased to 173 (35.2%) by visit 4. Similarly, the proportion of patients classified as “border line ill” increased to 261 (53%) at visit 4, while those classified as “mildly ill” showed a significant decrease to 51 (10.4%) at visit 4 (Fig. 8).

#### Change in Modified Simpson Angus Scale (MSAS)

The neurological side effects, specifically movement disorders, were evaluated using MSAS (Table 2). No neurological side effects were reported following the administration of pantoprazole and levosulpiride. Additionally, no patients exhibited a “clinically significant degree of movement disorders” or “severe degree of movement” abnormalities at visit 2, visit 3, or visit 4. A minimal degree of movement disorder was observed in one patient (0.2%) at visit 3, but was not deemed clinically relevant.

**Table 2:** Change in Modified Simpson Angus Scale (MSAS)

Statistics summary	Visit 2 (day 0) (N = 509)	Visit 3 (day 14 ± 2) (N = 507)	Visit 4 (day 28 ± 2) (N = 492)
Normal	508 (99.8%)	507 (100%)	492 (100%)
Minimal degree of movement disorder	1 (0.2%)	0 (0.0%)	0 (0.0%)
Clinically significant degree of movement disorder	0 (0.0%)	0 (0.0%)	0 (0.0%)
Severe degree of movement disorder is present	0 (0.0%)	0 (0.0%)	0 (0.0%)

## Discussion

The findings from this phase IV, open-label, single-arm, multicentric study provide supportive evidence for the short-term safety and efficacy of the FDC of pantoprazole and levosulpiride SR in patients with GERD who were unresponsive to PPI monotherapy. The observed clinical benefits over the 28-day treatment period suggest that this FDC may present a viable therapeutic alternative for a subset of GERD patients with partial or inadequate response to standard PPI therapy. However, the applicability of the results is limited to similar patient populations under comparable clinical settings. Although the multicentric approach enhances geographic and institutional diversity, the lack of a comparator arm limits conclusions regarding the relative efficacy of the FDC versus existing standard-of-care treatments. Consequently, the generalizability of these results to routine clinical practice should be interpreted with caution.

The study successfully met its primary endpoint, confirming the clinical safety of the FDC in the target population. All the reported AEs were as per the known profile of the drug. No SAEs or unexpected AEs were reported in the present study, and all the treatment-emergent adverse events (TEAEs) were mild to moderate in severity.

Hyperprolactinemia is also a well-documented effect of levosulpiride. The elevations in prolactin levels observed in our study are consistent with the known dopaminergic antagonism of levosulpiride and were not associated with any clinically significant manifestations during the study period. No patient was discontinued from the study due to hyperprolactinemia. In a study conducted by Lozano et al (2007), levosulpiride was given as 25 mg three times a day for 4 weeks, where galactorrhea was reported in 26.7% patients [21]. In another review by Mucci et al (1995), it was reported that in all clinical studies of levosulpiride, an increase of plasma prolactin concentration is frequent and this is not always associated with clinical manifestations [22]. These findings are also consistent with those of Kuchay and Mithal (2017), who reported significant prolactin elevations during levosulpiride therapy, without persistent AEs or SAEs [23]. Overall, the AEs observed in our study are consistent with the known pharmacological profile of the study drugs.

The observed safety profile is aligned with previously published data, including findings of Singhai et al (2016), who reported the absence of SAEs and no worsening of GERD symptoms following combination therapy with pantoprazole and levosulpiride. The observed safety profile in this study is consistent with established clinical data for the combination therapy of pantoprazole and levosulpiride [8, 21].

The most common AEs included headache, pyrexia, diarrhea, urinary tract infections, abdominal pain, constipation, oropharyngeal pain, dizziness, cough, nasopharyngitis, testicular pain, allergic rhinitis, nausea, and rhinitis. These findings align with previous research by Hein (2011), who reported that AEs associated with pantoprazole magnesium and pantoprazole treatment were predominantly gastrointestinal, such as diarrhea, vomiting, abdominal pain, and were mostly mild to moderate in severity [24]. Similarly, Pradeep Kumar et al (2017) also reported diarrhea, abdominal pain, and headache as the most frequently reported AEs with pantoprazole therapy [25].

The FDC of pantoprazole and levosulpiride demonstrated significant efficacy in reducing GERD symptoms, as assessed by validated scales like the FSSG Scale, Likert Scale, CGI-I, and CGI-S. In the present study, the FDC of pantoprazole and levosulpiride demonstrated a marked clinical benefit in patients with GERD, as evidenced by a 71.45% reduction in overall FSSG score. In particular, the mean FSSG score decreased significantly from 23.78 at baseline (visit 2) to 6.78 by visit 4, reflecting a substantial reduction in overall symptom burden. This improvement was further supported by a statistically significant mean (SD) change from baseline of −16.99 (8.76) (P < 0.0001), emphasizing the efficacy of the FDC of pantoprazole and levosulpiride in alleviating GERD symptoms. These findings align with those reported by Singhai et al (2016), who reported a 60.89% reduction in the mean FSSG score, from 18.88 to 8.28, following treatment with the FDC of pantoprazole and levosulpiride [8]. The slightly higher magnitude of response observed in the current study may be attributed to differences in baseline symptom severity, patient population, or duration of therapy [10]. Furthermore, a study conducted by Pradeep Kumar et al (2017) also reported a significant reduction in the GERD symptoms in the group treated with FDC of pantoprazole and itopride compared to pantoprazole monotherapy [25].

Furthermore, in the current study, acid reflux-related symptoms and dyspeptic symptoms showed statistically significant reduction by visit 4, reinforcing the clinical benefit of FDC of pantoprazole and levosulpiride in alleviating these symptoms in patients with GERD. These findings are consistent with those of a prospective study by Lakhtakia et al (2024), which demonstrated superior efficacy of FDC of pantoprazole and itopride compared to pantoprazole monotherapy, showing a significant reduction in acid reflux and overlapping dyspeptic symptoms in GERD patients [14]. Our findings align with prior reports supporting the role of prokinetics as adjuncts to PPIs. Additionally, an observational study by Wu et al (2019) explored the anti-reflux effects of pantoprazole combined with mosapride and domperidone in treating laryngopharyngeal reflux disease. The study reported superior symptom control with combination therapy comparted to PPI monotherapy, consistent with the observed benefits of prokinetic adjuncts in GERD management [26].

The Likert Scale assessment demonstrated statistically significant and progressive reduction in GERD symptom severity over the course of study. The mean symptom severity score declined from 2.55 at baseline to 1.28 by visit 4. The mean (SD) change from baseline was −1.26 (1.00) (P < 0.0001). Additionally, the proportion of patients reporting severe symptoms decreased markedly from 54.2% to 10% by visit 4. Notably, no patients reported very severe symptoms at the end of the study. These findings highlight the substantial symptom relief achieved with the FDC of pantoprazole and levosulpiride. The outcomes observed in the present study appear superior to those reported by Remes-Troche et al (2014) who evaluated the efficacy, safety, and tolerability of pantoprazole magnesium monotherapy in treating reflux symptoms in GERD patients, reporting up to an 80% reduction in symptom severity as assessed by the Likert Scale [27].

Improvement in overall patient condition was further substantiated by CGI-I and CGI-S assessments. In this study, 98.5% of patients showed improvement in GERD conditions in patient population, as measured by the CGI-I Scale by visit 4, and a significant reduction in disease severity measured by the CGI-S. These outcomes are comparable to those reported by Remes-Troche et al, (2014), who reported a 73% improvement in symptom intensity for GERD patients receiving pantoprazole [27]. Additionally, the CGI-I and CGI-S Scales provided reliable quantification of patient outcomes, emphasizing the efficacy of the treatment [28].

Notably, no movement disorder (neurological) side effects were observed, and all patients were assessed as normal at the end of the study. This may be attributed to the short duration of treatment as Shin et al (2009) reported that movement disorders typically develop after prolonged exposure to levosulpiride (12 to 27 months). The absence of such side effects in this study further supports the safety of short-term administration of the FDC, highlighting the duration-dependent nature of movement disorder risk and importance of treatment length in safety evaluations [29, 30]. Nevertheless, caution remains warranted, considering the known risk profile of levosulpiride, particularly with prolonged use.

Collectively, the findings from this study, supported by evidence from previous literature, underscore the clinical utility of the FDC of pantoprazole and levosulpiride as a safe and effective option for the short-term management of GERD.

### Limitations of the study

This study has limitations. The single-arm, open-label design limits the robustness of the findings, as it does not permit definitive causal inference, control for a placebo effect, or allow direct comparison with standard-of-care therapies. Treatment response was primarily assessed using validated symptom-based scoring systems and objective posttreatment evaluations such as repeat endoscopy or 24-h pH/pH-impedance monitoring were not included into the protocol. Although baseline endoscopy was performed, detailed characterization of findings (e.g., esophagitis grading, hiatal hernia, Barrett’s esophagus) and correlation with symptom response were not analyzed at predefined endpoints. The absence of ambulatory reflux monitoring also limits differentiation between pathological reflux, functional reflux, and hypersensitive esophagus. Refractory GERD was defined as persistence of typical symptoms despite at least 4 weeks of standard-dose PPI therapy. However, symptom predominance and prior disease duration were not formally stratified. The short treatment duration (28 days) restricts evaluation of long-term efficacy, recurrence after discontinuation, and sustained safety outcomes. No SAEs were observed during the study; however, levosulpiride carries potential neurological, endocrine, metabolic, and cardiac risks, particularly with prolonged use. Longer-duration, randomized controlled studies are warranted to confirm the long-term safety and therapeutic sustainability.

### Conclusion

The study demonstrated that an FDC of pantoprazole and levosulpiride was safe and effective for the short-term treatment of GERD in patients unresponsive to the PPI monotherapy. The substantial reduction in symptom severity, along with a manageable AE profile, highlights the clinical utility of this combination therapy. Future investigations employing randomized, controlled designs, with extended follow-up periods and broader inclusion criteria are warranted to explore the long-term safety, sustained efficacy, and potential role of this FDC in the broader landscape of evolving GERD management strategies.

## Data Availability

The data supporting the findings of this study can be obtained from the corresponding author upon reasonable request.

## Abbreviations

AEs: adverse events
ASPR: age-standardized prevalence rate
BMI: body mass index
CGI-I: Clinical Global Impression of Improvement
CGI-S: Clinical Global Impression of Severity
CTRI: Clinical Trials Registry-India
FDC: fixed-dose combination
FSSG: Frequency Scale for the Symptoms of GERD
GERD: gastroesophageal reflux disease
ICH-GCP: International Council for Harmonisation’s-Good Clinical Practice guidelines
IEC: Institutional Ethics Committee
LES: lower esophageal sphincter
MSAS: Modified Simpson Angus Scale
NSAIDs: nonsteroidal anti-inflammatory drugs
pH: power of hydrogen
PPIs: proton pump inhibitors
SAEs: serious adverse events
SD: standard deviation
SR: sustained release
SAS: Statistical Analysis System
TEAEs: treatment-emergent adverse events
USA: United States of America
